# Phase diagram of hard squares in slit confinement

**DOI:** 10.1038/s41598-018-26922-3

**Published:** 2018-06-11

**Authors:** Gustavo Bautista-Carbajal, Péter Gurin, Szabolcs Varga, Gerardo Odriozola

**Affiliations:** 1 Academia de Matemáticas, Universidad Autónoma de la Ciudad de México, 07160 México, Distrito Federal Mexico; 2 Institute of Physics and Mechatronics, University of Pannonia, P.O. Box 158, Veszprém, H-8201 Hungary; 3 Área de Física de Procesos Irreversibles, División de Ciencias Básicas e Ingeniería, Universidad Autónoma Metropolitana-Azcapotzalco, Av. San Pablo 180, 02200 CD México, Mexico

## Abstract

This work shows a complete phase diagram of hard squares of side length *σ* in slit confinement for *H* < 4.5, *H* being the wall to wall distance measured in *σ* units, including the maximal packing fraction limit. The phase diagram exhibits a transition between a single-row parallel 1-${\boldsymbol{\square }}$ and a zigzag 2-$\hat{\diamond }$ structures for *H*_*c*_(2) = (2$\sqrt{{\bf{2}}}$ − 1) < *H* < 2, and also another one involving the 1-${\boldsymbol{\square }}$ and 2-${\boldsymbol{\square }}$ structures (two parallel rows) for 2 < *H* < *H*_*c*_(3) (*H*_*c*_(*n*) = *n* − 1 + $\sqrt{{\bf{2}}{\boldsymbol{n}}-{\bf{1}}}$/*n* is the critical wall-to-wall distance for a (*n* − 1)-${\boldsymbol{\square }}$ to *n*-${\diamond }$ transition and where *n*-${\diamond }$ represents a structure formed by tilted rectangles, each one clustering *n* stacked squares), and a triple point for *H*_*t*_ ${\boldsymbol{\simeq }}$ 2.005. In this triple point there coexists the 1-${\boldsymbol{\square }}$, 2-${\boldsymbol{\square }}$, and 2-$\hat{\diamond }$ structures. For regions *H*_*c*_(3) < *H* < *H*_*c*_(4) and *H*_*c*_(4) < *H* < *H*_*c*_(5), very similar pictures arise. There is a (*n* − 1)-${\boldsymbol{\square }}$ to a *n*-${\diamond }$ strong transition for *H*_*c*_(*n*) < *H* < *n*, followed by a softer (*n* − 1)-${\boldsymbol{\square }}$ to *n*-${\boldsymbol{\square }}$ transition for *n* < *H* < *H*_*c*_(*n* + 1). Again, at *H* ${\boldsymbol{\gtrsim }}$ *n* there appears a triple point, involving the (*n* − 1)-${\boldsymbol{\square }}$, *n*-${\boldsymbol{\square }}$, and *n*-${\diamond }$ structures. The similarities found for n = 2, 3 and 4 lead us to propose a tentative phase diagram for *H*_*c*_(n) < *H* < *H*_*c*_(n + 1) (n ∈ ${\boldsymbol{{\mathbb{N}}}}$, *n* > 2), where structures (*n* − 1)-${\boldsymbol{\square }}$, *n*-${\boldsymbol{\square }}$, and *n*-${\diamond }$ fill the phase diagram. Simulation and Onsager theory results are qualitatively consistent.

## Introduction

Athermal systems, despite their apparent simplicity, often show rich behaviour. Playing with particle’s shape and confinement leads to a huge variety of self-assembling structures^1–9^. Arrangement usually happens at nano and mesoscale levels, driven by thermal fluctuations, but also at macroscopic scales, where container twists^10^ or tapping movements^11^ are applied to play the role of thermal fluctuations. On the one hand, particles’ shape induce directional entropic forces which may drive self assembling into anisotropic phases^5,12^. On the other hand, confinement introduces frustration which may force the system to arrange into complex structures^3,9,10,13–16^, and to follow unusual phase transitions^17–20^. Hence, it comes as no surprise that their combination results in colourful phase diagrams, and even in the appearance of anomalous behaviours^6,21–25^. The thermodynamic equilibrium of such systems is, often, non-trivial. In addition, predicting the equilibrium phase for confined systems is important not only from theoretical but also from practical purposes, given the actual exquisite experimental control of shape and size of nano and micro sized colloids^5,26,27^, and the existence of lithographic, layer by layer, and template-directed growth^6,28,29^, which makes possible the bottom-up approach for new materials design.

**Figure 1:**
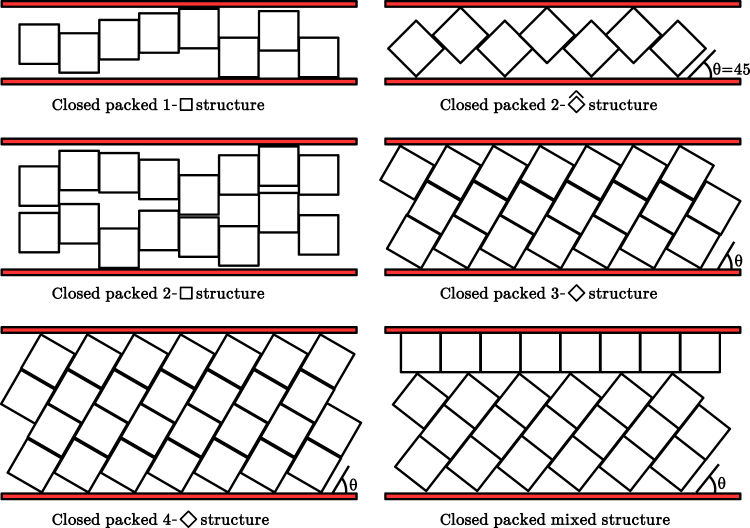
Competing closed packed structures for different confinement distances, *H*, as labelled. The x and y directions are defined along to and perpendicular to the channel, respectively.

Decreasing the dimensionality of the system does not always lead to a simpler behaviour. For instance, the freezing of hard discs in a two dimensional plane is by far more complicated than the freezing of their three dimensional analogue (hard spheres). Indeed, the fluid-hexatic, hexatic-solid complex transition has been extensively debated^15,30–33^. In principle, going down to one dimensional systems makes things easier, since there appear a relatively large number of model systems which are analytically solvable. In addition, the van Hove’s theorem^34,35^ rules out the existence of genuine thermodynamic transitions (the free energy and all its derivatives are continuous at the thermodynamic limit) for short range potentials. Nonetheless, there exists some systems which exhibit peculiar behaviours at high pressures^36^. In our recent works^24,25^, we have found that quasi-one dimensional confined hard squares of side length *σ* show a strong structural transition involving the structures given at the top line of Fig. 1 in the range $(2\sqrt{2}-1)={H}_{c}(2) < H < 2$, *H* being the wall-to-wall distance measured in units of *σ*. We have also found that the packing fraction, *η*, versus pressure along the channel, *P*_*x*_, shows a step function like behaviour resembling a true discontinuity. Moreover, the transition strengthens with decreasing *H*, becoming critical in the limit *H* → *H*_*c*_(2) and *P*_*x*_ → ∞, with critical exponents belonging to the universality class of the one-dimensional Ising model^25^. Although there is no critical point at any finite pressure, there is a real critical behaviour (which can be experimentally observable) in the vicinity of the critical point. As a result, simulations (or any real experiment composed by a very large but finite number of particles) are not capable of distinguishing this extremely sharp structural transition from a genuine thermodynamic one.

In this work, by mainly focusing on simulations, we extend previous results to build the phase diagram for a larger *H* region, expanding up to *n* = 4 layers of particles through the channel. For this purpose we first extend the maximal packing fraction curve, as a function of *H*, by assuming that only the parallel and tilted structures lead to the maximal packing fractions (examples of these structures are depicted in Fig. 1). We give details of this proposal in the next section. Next, we present the simulation methods and their results in the following two sections, respectively. In this last section we focus on the building of the phase diagram. We first summarise the previous results for *H*_*c*_(2) < *H* < 2, where the 1-$\square$ to 2-$\hat{\diamond }$ structural transition appears, and extend the study to the region 2 < *H* < *H*_*c*_(3), where a soft transition is found involving the 1-$\square$ and 2-$\square$ structures. Curiously, the 2-$\hat{\diamond }$ structure persists only for *H* values above but very close to 2, and a triple point emerges at ${H}_{t}\simeq 2.005$ where the three structures coexists. This picture seems to be replicated to the *H*_*c*_(3) < *H* < *H*_*c*_(4) and *H*_*c*_(4) < *H* < *H*_*c*_(5) regions, being the only difference that the zigzag structure, 2-$\hat{\diamond }$, is replaced by a tilted one having *n* = 3, 4 layers. Also, the strength of the (*n* − 1)-$\square$ to the *n*-${\diamond }$ transition increases with *n*, and the triple point shifts slightly to higher *H*. The strong similarities encourages us to propose an approximate phase diagram for a general region *H*_*c*_(*n* − 1) < *H* < *H*_*c*_(*n*).

## Maximal Packing Fraction

As mentioned in the introduction, the 1-$\square$ and 2-$\hat{\diamond }$ structures are those leading to the highest possible close packing for *H* < 2. The close packing fraction of the first one is simply

1 $${\eta }_{cp}^{\square }(H)={H}^{-1},$$

where we recall that *H* is given in units of the side length of the square, *σ*. In general, for *n*-$\square$ structures we have

2 $${\eta }_{cp}^{\square }(n,H)=n{H}^{-1}.$$

On the other hand, the close packing fraction of the 2-$\hat{\diamond }$ zigzag structure is

3 $${\eta }_{cp}^{\hat{\diamond }}(H)={[H(2\sqrt{2}-H)]}^{-1},$$

which is increasing in the range $\sqrt{2} < H < 2$. These two close packing fractions intersect at ${H}_{c}(2)=2\sqrt{2}-1$, which coincides with the location of the critical point for the 1-$\square$ to the 2-$\hat{\diamond }$ structural transition^25^.

For larger *H* values, we find that the *n*-${\diamond }$ structure competes with the (*n* − 1)-$\square$ structure and that yields the densest packing for *H*_*c*_(*n*) < *H* < *n* (examples of these structures are depicted in Fig. 1). As already mentioned, this former structure is composed by tilted rectangles of *n* stacked squares. Its close packing fraction is given by

4 $${\eta }_{cp}^{\diamond }(n,H)=\frac{n\,\sin \,\theta }{H},$$

*θ* being the angle between the large side of the rectangle and the direction along the channel (see Fig. 1). The value of *θ* can be determined from the equation $n\,\sin \,\theta +\,\cos \,\theta =H$. In the investigated region, *n* − 1 ≤ *H* < *n* has a unique solution in the *θ* ∈ [0, *π*/2] interval, which is given by

5 $$\sin \,\theta =\frac{Hn-\sqrt{{n}^{2}+1-{H}^{2}}}{{n}^{2}+1}.$$

**Figure 2:**
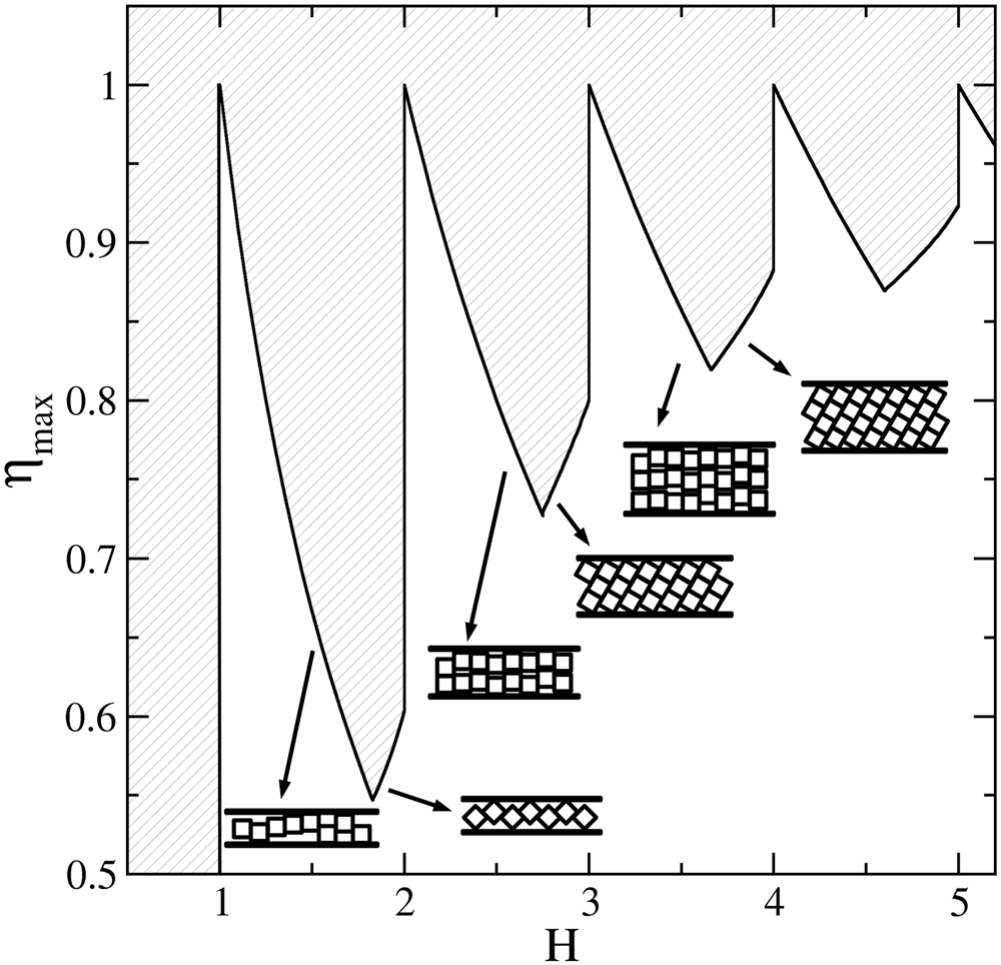
Maximal packing fraction *η*_*max*_ as a function of the separation distance, *H*. Hatched areas are inaccessible to the system.

Note that ${\eta }_{cp}^{\diamond }$(*n*, *H*) is, as ${\eta }_{cp}^{\hat{\diamond }}$(*H*), an increasing function of *H* in its corresponding *H* range. Again, the point at which ${\eta }_{cp}^{\square }(n-1,H)={\eta }_{cp}^{\diamond }$ (*n*, *H*) defines *H*_*c*_(*n*), coinciding with the presumably critical point for the (*n* − 1)-$\square$ to the *n*-${\diamond }$ transition. In turn, *H*_*c*_(*n*) is given by

6 $${H}_{c}(n)=n-1+\sqrt{2n-1}/n,$$

which is always in the *n* − 1 < *H*_*c*_(*n*) < *n* range, and tends to be *n* − 1 for large *n*. Hence, for increasing *n*, the range at which the n-${\diamond }$ structure produces the maximal packing fraction widens (see Fig. 2).

Note that for wide pores one may expect to find hybrid structures having a mixture of *m* rows parallel to the walls, and tilted rectangles composed by *n* − *m* squares (an example with *n* = 4 and *m* = 1 is depicted at the right hand side and bottom of Fig. 1). In this case we would have

7 $${\eta }_{cp}^{mix}(n,m)=m/H+(H-m){\eta }_{cp}^{\diamond }(n-m,\theta (H-m))/H$$

whenever the *m* rows are wetting one  or both of the confining planes, but not dispersed at bulk which would lead to a less efficient packing. It can be shown that this packing fraction is a decreasing function of *m*, and is always below ${\eta }_{cp}^{\square }(n-\mathrm{1)}$ for *n* − 1 < *H* < *H*_*c*_(*n*) and below ${\eta }_{cp}^{\diamond }$(*n*) for *H*_*c*_(*n*) < *H* < *n*. Note that angle *θ* decreases with decreasing *H*, which yields a less efficient packing. Nonetheless, the difference ${\eta }_{cp}^{\diamond }(n)-{\eta }_{cp}^{mix}(n,m)$ tends to zero for n → ∞ and finite *m*.

## Simulation Method

As in previous work^24^, we are employing the replica exchange Monte Carlo technique to sample from equilibrium, whenever possible. This technique enhances the natural trend of the Monte Carlo method to reach equilibrium when the free energy landscape is wrinkled or simply split by hills (our case). The technique rests on the definition of an extended ensemble, ${Q}_{ext}={\prod }_{i}\,{Q}_{i}$, where *Q*_*i*_ represents the partition function of three macroscopic thermodynamic variables, being them functions of *i*^37–40^. Its most popular implementation involves the temperature expansion of the canonical ensemble, ${Q}_{ext}={\prod }_{i}\,Q(N,V,{T}_{i})$, frequently referred to as parallel tempering^37^. Of course, in our case this implementation has no benefit at all, due to the hard character of our inter-particle potential. Instead, we are employing ${Q}_{ext}={\prod }_{i}\,Q(N,{P}_{x,i},T)$, that is, a pressure expansion of the isobaric ensemble (employing the pressure component along the channel)^41^. This form has been successfully employed to determine the phase diagram of several hard systems^42–44^, even when dealing with phase transitions between very dense phases such as solids^42^.

As mentioned, our system consists of a collection of *N* identical squares placed in a plane (a two dimensional system), and confined by two parallel walls (lines). Walls are separated by a distance *H* the one from the other (measured in units of *σ*, the side length of our squares), and placed parallel to our *x* direction. A second *y* axis is defined perpendicular to the *x* axis and inside the system plane. Periodic boundary conditions are set only for the *x* axis. Verlet lists are implemented to gain efficiency. Note that the gain is huge for small *H* values, where lists are rarely refreshed. Indeed, for *H* < 2 each particle has only two fixed neighbors. The sampling of each *Q*(*N*, *P*_*x*,*i*_, *T*) subensemble is done by implementing a standard isobaric sampling. This sampling involves particle displacements, particle rotations, and changes of the length of the *x* side of the channel (area changes with a fixed *H*). We are setting 32 system replicas (unless otherwise indicated), each one located at a corresponding *P*_*x*,*i*_. After some *NP*_*x*_*T* cycles, we stop the MC threads and proceed to trial some replica interchanges. These trials attempt to swap a couple of randomly selected replicas, located at different but adjacent pressures. The acceptance probability for swap trials reads ${\rm{\min }}\,\{1,\exp [\beta ({P}_{x,i}-{P}_{x,j})\,({A}_{i}-{A}_{j})]\}$, where *A*_*i*/*j*_ and *P*_*x*,*i*/*j*_ are the area and longitudinal pressure of replica *i*/*j*. Immediately after, the standard MC threads are restarted defining an external cycle. This overall cycle is repeated a very large number of times, as much as necessary.

**Figure 3:**
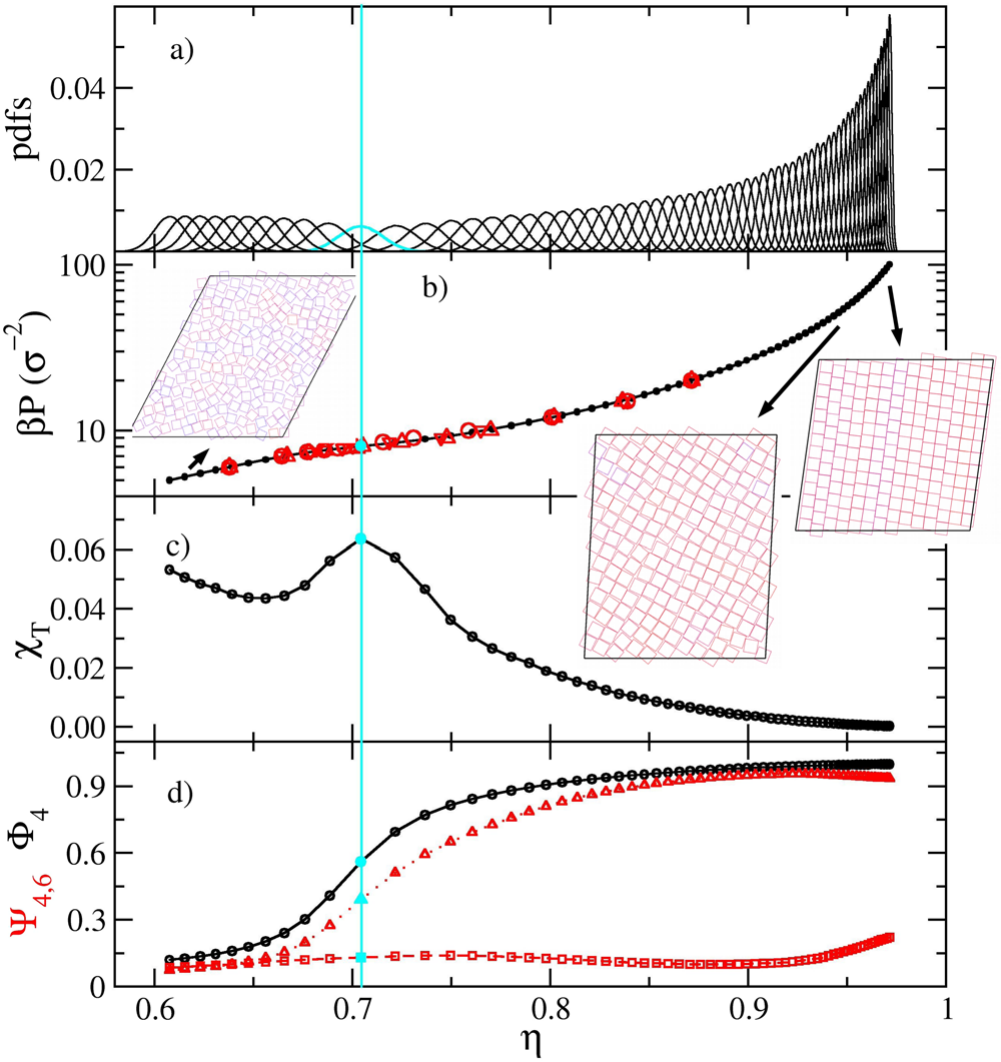
Simulation results for unconfined squares. (**a**) Probability density functions. (**b**) Pressure as a function of the packing fraction. Open circles correspond to reference^45^, and triangle-ups and triangle-down symbols to reference^22^ for compression and expansion, respectively. (**c**) Dimensionless isothermal compressibility. (**d**) 4-fold orientation order parameter Φ_4_, and 4 and 6-fold bond order parameters, Ψ_4_ and Ψ_6_, respectively. The light (cyan) vertical line highlights the approximate position of the isotropic fluid-solid two step soft transition. The tetratic phase appears in a small density window close to this point. The insets are snapshots corresponding to the regions pointed out by the arrows.

To test our implementation we proceed to turn off the confinement walls, turn on the *y* periodic boundary condition, turn on angle and size changes of the simulation cells (important to gain degrees of freedom and avoid artefacts due to geometric frustration), and sample a bulk of *N* = 196 squares with 64 replicas (corresponding to the 64 points of Fig. 3). The outcome is presented in Fig. 3, where we have included data points from references^45^ and^22^ for the equation of state. We are also adding the isothermal compressibility *χ*_*T*_ = *N*(〈*ρ*^2^〉 − 〈*ρ*〉^2^)/〈*ρ*〉^2^, *ρ* = *N*/*A* being the number density, and order parameters. These lasts read ${{\rm{\Phi }}}_{4}=\frac{1}{N}|{\sum }_{j=1}^{N}\,\exp \,(4i{\theta }_{j})|$ and ${{\rm{\Psi }}}_{n}=\frac{1}{N}|{\sum }_{j=1}^{N}\,{\frac{1}{n}}_{j}\,{\sum }_{k=1}^{{n}_{j}}\,\exp \,(ni{\theta }_{jk})|$, Φ_4_ being the 4-fold order parameter and Ψ_*n*_ the *n*-fold bond order parameter. In these expressions *θ*_*j*_ is the angle formed by any of the sides of the square *j* and an arbitrary reference direction, *n*_*j*_ is the number of bonding particles to square *j*, and *θ*_*kj*_ are the angles between the line connecting the centres of squares *k* and *j* and another arbitrary direction (for simplicity, the same reference is taken). The very good agreement found among the independent simulations points out the correctness of our unconfined code, where the isotropic fluid to the solid phase transition is confirmed smooth, and where the tetratic phase appears in-between them^45^. This result is also consistent with Anderson *et al*. recent long scale simulations, where a Kosterlitz-Thouless-Halperin-Nelson-Young^30,31^ smooth two-step transition is found for the melting of squares^46^. Note that the bell-shaped probability density functions widen and become lower at the transition keeping their Gaussian profile, never distorting into bimodals. At high pressures, we observe a small decrease of Ψ_4_ accompanied with a small increase of Ψ_6_, suggesting the existence of few replicas exhibiting well defined rows, ones shifted with respect to the others (see the snapshot inset in Fig. 3). This feature has been, to our knowledge, not previously reported. The code accounting for confinement also produces results consistent with the exact results of the transfer matrix method^24,25^.

We should also add here that perfect squares with sharp edges are difficult to obtain in the lab, and in general, sharp edges do not frequently occur in nature. In particular, Zhao *et al*. have built, by lithography, 2D systems of Brownian hard squares with tiny roundness at their corners^6^. They have osmotically controlled the area fraction to study the phase behaviour of a monolayer. Strikingly, they have found no tetratic nor square crystals at all. Instead, they have found a transition from an isotropic fluid to a rhombic crystal, passing trough a hexagonal rotator phase, neither of them showing the expected four-fold symmetry of the constituting particles. They capture the general behaviour by employing a simple Onsager description and conjectured that the tiny roundness around the corners of their squares were the source of the dramatic change of phase behaviour of the system. This was then corroborated by Avendaño and Escobedo, who explore with MC the phase behaviour of squares with different degrees of roundness^22^. In addition, they even reported for a certain degree of roundness a polycrystalline phase with domains of square order in coexistence with clusters showing weak hexagonal order. This nice story constitutes an iconic example of the key role of shape in the thermodynamics of hard systems, but it is not the only one^46^.

Back to our confined system of perfect squares, we start the simulations from loose random configurations and run the code until a steady state is observed (unless otherwise indicated). Unfortunately, this steady state does not always correspond to equilibrium. This is particularly true when dealing with the (*n* − 1)-$\square$ to *n*-${\diamond }$ transition. In the simulation we observe a coexistence of these structures even in the case of *n* = 2, where such a thing is ruled out by theory. For this kind of coexistence we start simulations with *N* ≈ 100 from cells filled with both competing structures, each having the same number of particles, and a couple of boundaries. For a replica set with a small *P*_*x*_ value, the (*n* − 1)-$\square$ structure grows at the expenses of the *n*-${\diamond }$ one. Conversely, the *n*-${\diamond }$ structure tends to grow (with difficulty) for *P*_*x*_ above the transition (coexistence) value. This way, when reaching a steady state, we can find an approximate value of the transition pressure. Once this pressure is obtained, we simply run the code by starting from cells having pure (*n* − 1)-$\square$ or *n*-${\diamond }$ structures, located below and above the transition pressure, respectively. From these last simulations we get the transition densities depicted as red bullets in the phase diagrams for the (*n* − 1)-$\square$ to *n*-${\diamond }$ transition. The (*n* − 1)-$\square$ to *n*-$\square$ smooth transition is signalled by a single red bullet which corresponds to the maximum of the isothermal compressibility.

## Results

**Figure 4:**
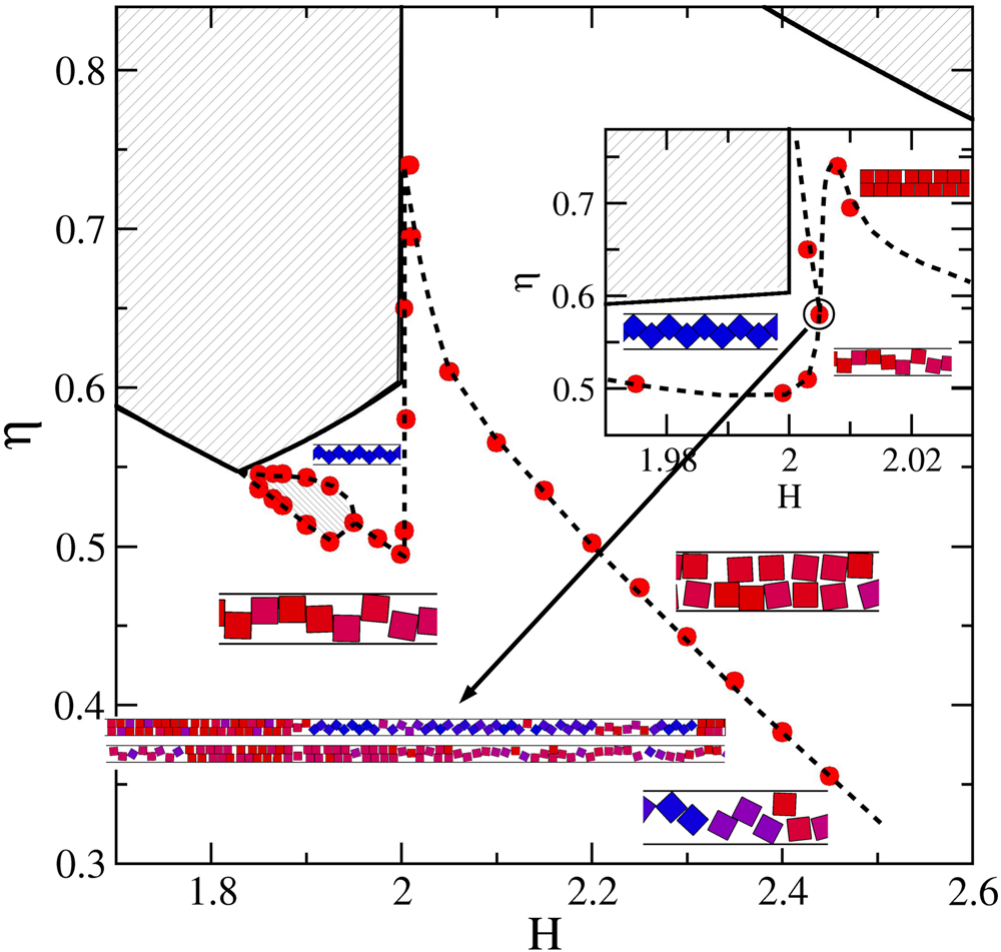
Phase diagram for the *H*_*c*_(2) < *H* < *H*_*c*_(3) region. Red bullets are data from simulations. The inset zooms in the *H* ≈ 2 region. Dashed lines are guides to the eye. Lightly hatched areas are inaccessible. The heavy hatched area points out a transition region in which *P*_*x*_ practically does not depend on *η*. The small snapshots are sections of the simulation cells, which are located according to their *η* and *H* values. The long snapshots pointed by an arrow correspond to cells appearing at the triple point. This point is highlighted by a circle. In the snapshots, parallel and 45-tilted squares are coloured red and blue, respectively. Intermediate angles are painted with a mixture of both colours.

We first focus on the *H*_*c*_(2) < *H* < *H*_*c*_(3) region of the phase diagram (see Fig. 4), for which we take advantage of the data previously published for *H*_*c*_(2) < *H* < 2^24^. The region where structures 1-$\square$ and 2-$\hat{\diamond }$ (the zigzag structure) compete has been solved employing both, simulations and theoretical calculations^24,25^. There we have observed that structure $\hat{\diamond }$ is preferred at high pressures and high packing fractions, and the 1-$\square$ structure is obtained at lower pressures. Note that the particles of the 1-$\square$ structure have more room for fluctuations in the transversal direction than those of the 2-$\hat{\diamond }$ structure. Therefore the particles stay in 1-$\square$ structure at low densities even if the 2-$\hat{\diamond }$ one can be more packed. This explains our findings. In addition, we have found that at the vicinity of $H\lesssim 2$, the structural transition is smooth. However, the transition strength dramatically grows for decreasing *H*, which can be seen in sharper and higher peaks in the isothermal compressibility (see refs^24,25^). By observing the simulation results only, we could not discard a genuine first order transition for $H\lesssim 1.9$. That is, the gap between the phases (let us call them phases) turns really obvious, a kind of one dimensional bubbles grow large, the dimensionless isothermal compressibility yield high and narrow peaks which increases with system size, and structural order parameters abruptly change, being all these features hallmarks of true first order transitions. Indeed, we have found that a discretized version of this system^25^, which can be solved analytically, has a critical point at (*H*_*c*_(2), *P*_*x*_ → ∞), and exhibits a critical behaviour at its neighbourhood, corresponding to the universality class of the one-dimensional Ising model. We have also shown that this simplified (orientational and y-positional restricted) version captures the key features of the unrestricted system and so, we also expect the freely rotating squares to show the same critical behaviour at the vicinity of (*H*_*c*_(2), *P*_*x*_ → ∞). Hence, although we know from theoretical considerations that the transition is not genuine, we are pointing out in Fig. 4 a transition region in which the pressure, *P*_*x*_, is practically independent of the packing fraction, *η*. From the point of view of simulations only, this region is indistinguishable from a coexistence region. We are also, from time to time, naming the competing structures as phases.

**Figure 5:**
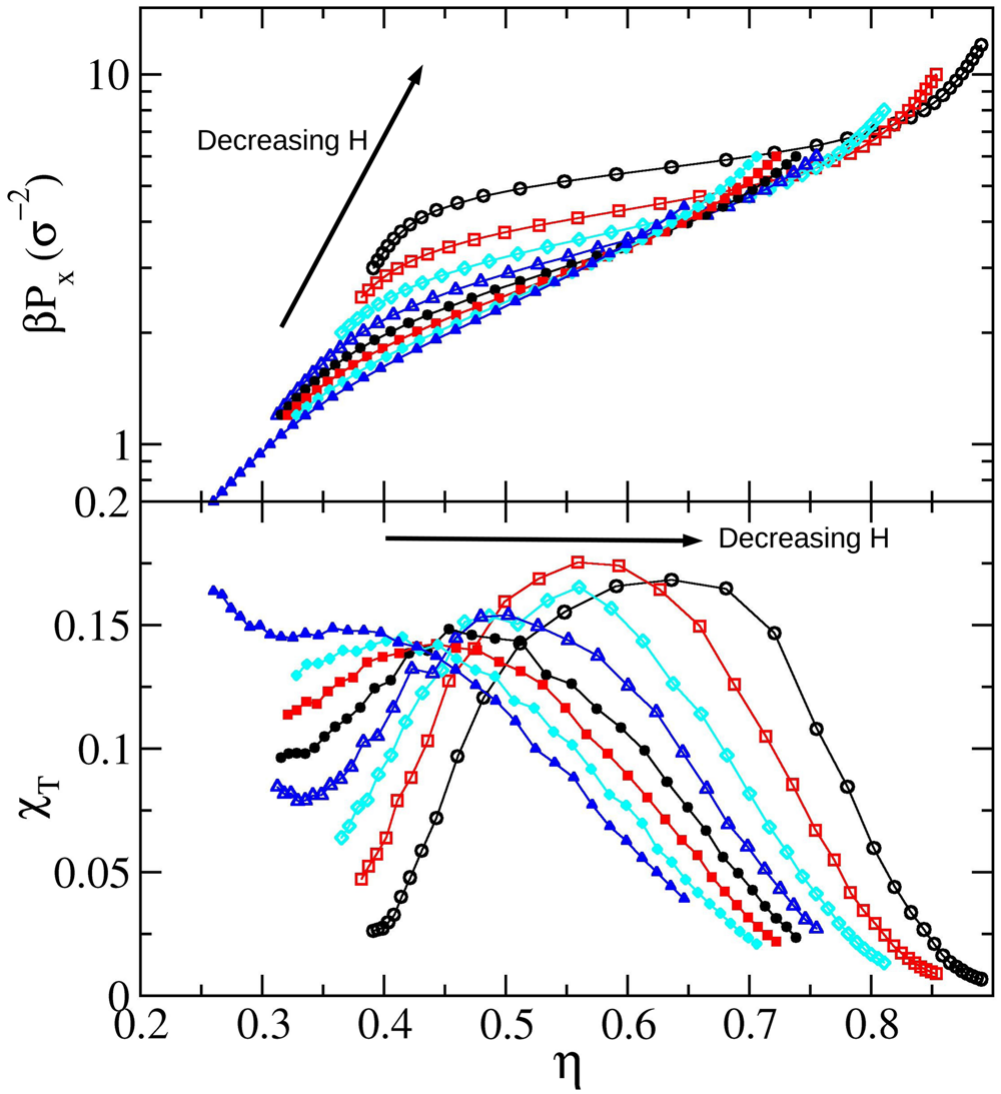
Equations of state (top) and dimensionless isothermal compressibility (bottom) for different wall-to-wall separation distances, *H*. *H* increases as shown by the inserted arrows. The covered range is 2.05 ≤ *H* ≤ 2.4, with increments of 0.05.

To build up the *H* > 2 region of Fig. 4, we firstly conduct *N* = 500 (considering 32 replicas) simulations covering the range 2.05 ≤ *H* ≤ 2.40 by intervals of 0.05. These simulations are started from loose random cells. Results are given in Fig. 5 where a smooth and wide layering transition is observed, involving the 1-$\square$ and 2-$\square$ structures. Note that as *H* increases, the 1-$\square$ structure becomes more fluid-like making the single row to fade. Hence, what we called the 1-$\square$ structure can be strongly different for low and high *H* values. It is worth mentioning that the finding of a 2-$\square$ structure for *H* > 2 is consistent with our proposal of having the 2-$\square$ structure as that producing the maximal packing fraction in this *H* region. From Fig. 5, we are taking the compressibility maximum as the point at which the transition takes place. The obtained transition points are then depicted as red bullets in Fig. 4. The transition never exhibits signatures of a first order one and its strength decreases for increasing *H*. In fact, we cannot detect a clear compressibility peak for *H* > 2.40, though a smooth structural change remains observable. From Fig. 5 it is clear that the position of the compressibility peaks shifts to larger *η* values for decreasing *H*. The shifting, in turn, strengthens when approaching *H* = 2, in such a way that the observed trend yields relatively high pressure and density values for *H* → 2 (see Fig. 5). At this point, the emerging phase diagram would consist of exclusively having the 1-$\square$ and 2-$\square$ structures in the interval 2 < *H* < *H*_*c*_(3), since we never detect the appearance of the 2-$\hat{\diamond }$, even for *H* = 2.05. This made us thought, for some moment, that the 2-$\hat{\diamond }$ would suddenly disappear at *H* = 2, which would be a somewhat peculiar behaviour. It turned out that the complete real picture is probably even more peculiar, involving a triple point.

**Figure 6:**
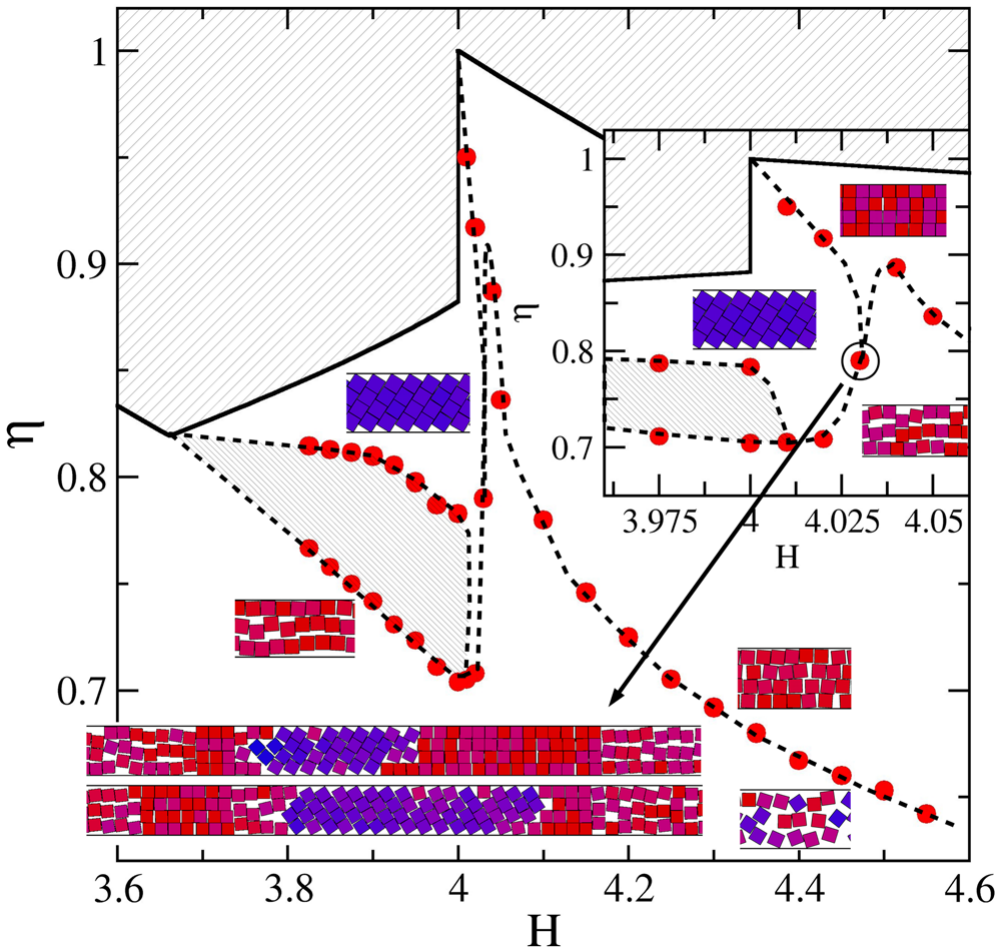
Phase diagram for the *H*_*c*_(4) < *H* < *H*_*c*_(5) region. Red bullets are data from simulations. The inset zooms in the *H* ≈ 4 region. Dashed lines are guides to the eye. Lightly hatched areas are inaccessible. The heavy hatched area points out a transition region, in which *P*_*x*_ practically does not depend on *η*. The small snapshots are sections of the simulation cells, which are located according to their *η* and *H* values. The long snapshots pointed by an arrow correspond to cells appearing at the triple point. This point is highlighted by a circle. In the snapshots, parallel and 45-tilted squares are coloured red and blue, respectively. Intermediate angles are painted with a mixture of both colours.

Before entering into the details of the tiny region around *H* = 2, we performed simulations on the *H*_*c*_(4) < *H* < *H*_*c*_(5) domain. Results are given in Fig. 6. There we have found a very similar behaviour than for the *H*_*c*_(2) < *H* < *H*_*c*_(3) case, being the main difference that the 2-$\hat{\diamond }$ structure (the zigzag structure) is replaced by the 4-${\diamond }$ structure, composed by parallel rectangles made up of, in this case, 4 stacked squares. Similarities are remarkable. On the one hand, for *H*_*c*_(4) < *H* < 4, there is a 3-$\square$ 4-${\diamond }$ transition region close to *H*_*c*_(4). The strength of this transition increases for *H* approaching *H*_*c*_(4), and the pressure seems to diverge at this point. The transition region, as for the *H*_*c*_(2) < *H* < *H*_*c*_(3) case, seems to abruptly end at *H*_*c*_, making the (*H*_*c*_, *P*_*x*_ → ∞) point to strongly resemble a critical point. Let us call it that way, despite we have not characterised its likely critical behaviour. This task may yield critical exponents different than those obtained for the *H*_*c*_(2) < *H* < *H*_*c*_(3) case, given the gain of fluctuations in the *y*-axis direction. On the other hand, for 4 < *H* < *H*_*c*_(5) we have found a very similar layering transition than the one found for the 1-$\square$ 2-$\square$ structures, but now involving the 3-$\square$ 4-$\square$ structures. In this case, we clearly detect peaks of *χ*_*T*_ up to *H* = 4.5, then the transition turns too soft and peaks vanish. As before, the transition shifts to larger *η* and *βP*_*x*_ values and strengthens for decreasing *H*, but keeping always a soft behaviour. We note that a similar layering transition has been also observed in a system of hard rectangles confined between parallel lines or in rectangular cavities^47–49^. Again, note that as *H* increases, the 3-$\square$ structure turns fluid-like where well defined rows tend to disappear.

**Figure 7:**
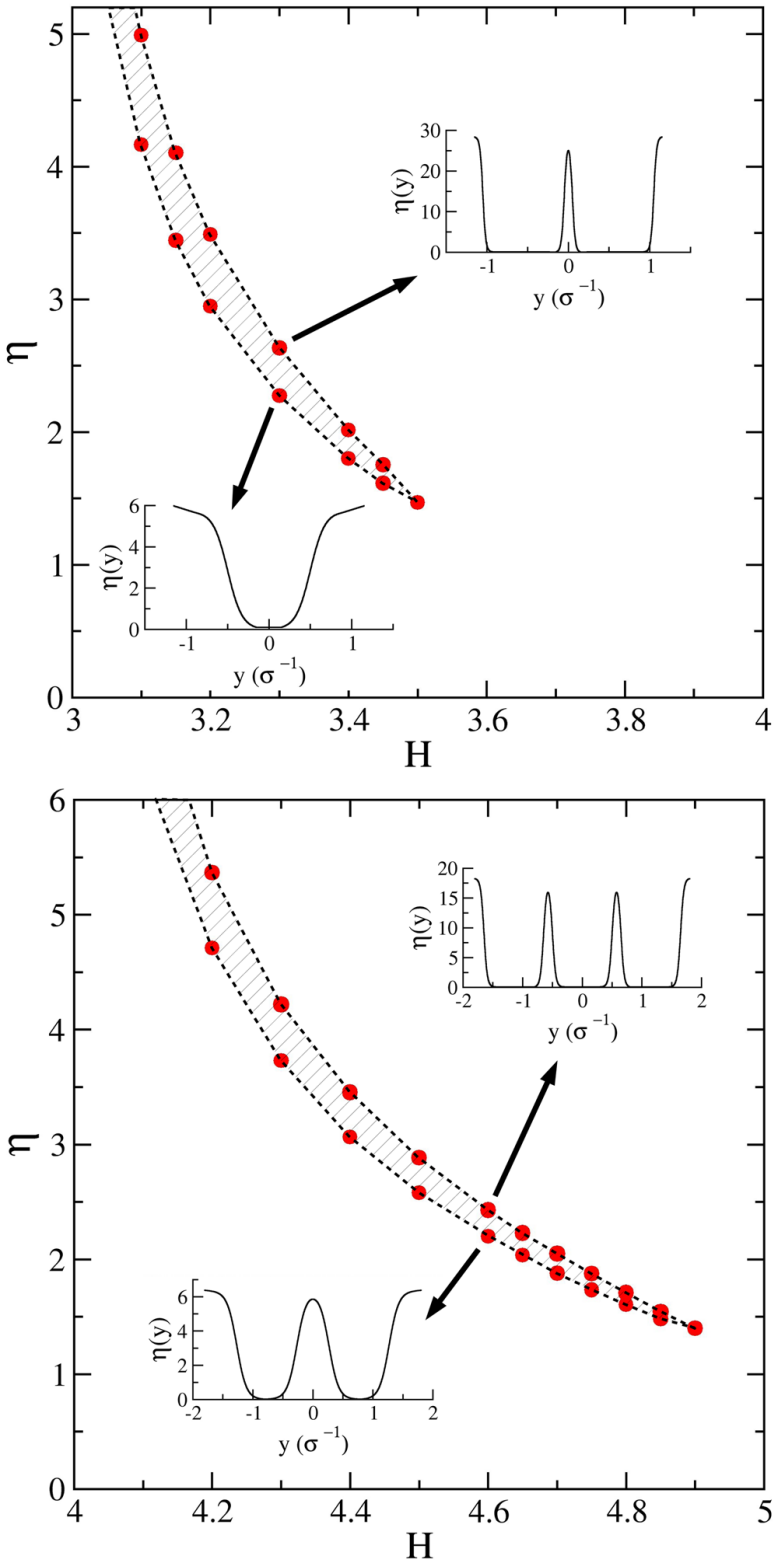
Layering transition diagram as obtained from the Onsager second virial theory and by considering a restricted system of squares with a couple of sides parallel to the walls. The top and bottom panels depict the 3 < *H* < 4 and the 4 < *H* < 5 regions, respectively. The insets show the density profiles as obtained at the (*H*,*η*) points signalled by the arrows. The drawings schematise the corresponding system configuration. The hatched areas point out a transition region in which *P*_*x*_ does not depend on *η*.

The layering phenomenon between the *n*-$\square$ and (*n* + 1)-$\square$ structures (fluids with *n* and *n* + 1 layers) can be understood on the basis of Onsager second virial theory, too^50^. Considering the system of parallel hard squares between two hard walls, i.e. the effect of orientational freedom is discarded, the free energy density can be written as a sum of an ideal gas contribution, plus an excess and external field terms as follows,

8 $$\frac{\beta F}{{L}_{x}}=\int \,dy\,\rho (y)\,(\rho (y)-1)+\frac{1}{2}\,\int \,d{y}_{1}\,\rho ({y}_{1})\,\int \,d{y}_{2}\,\rho ({y}_{2}){d}_{{\rm{exc}}}({y}_{12})+\int \,dy\,\rho (y)\beta {V}_{{\rm{ext}}}(y)$$

where *L*_*x*_ is the length of the system along the *x* axis, *ρ*(*y*) = *η*(*y*)/*σ*^2^ is the local density, *d*_*exc*_(*y*_12_) = 2*σθ*(*σ* − |*y*_12_|) is the excluded distance between two squares at positions *y*_1_ and *y*_2_, respectively, *y*_12_ = *y*_1_ − *y*_2_, and *V*_ext_(*y*) is the external potential acting on a particle at position *y*. Certainly, the local density can be higher than zero only inside the pore, because *V*_ext_ = 0 for *y* ≤ |*H* − *σ*|/2 and *V*_ext_ = ∞ for *y* > |*H* − *σ*|/2. The first term of the free energy (proportional to translational entropy) favours the homogeneous local density, the second one (packing entropy) wants to maximise the available room for the particles, and the last one constrains the particles to be inside the pore. To find the equilibrium density profile at a given packing fraction and *H*, the free energy must be minimised with respect to the local density with the condition of $\eta ={\sigma }^{2}{H}^{-1}\,\int \,dy\,\rho (y)$. The details of such calculations can be found elsewhere^50^. At very low packing the ideal gas term wins with almost constant density profile because the contribution of *d*_exc_ is negligible. The situation changes dramatically with increasing *η*, since the packing is the best for particles aligning in the same row and the contribution of *d*_exc_ becomes dominant. As a result of the competition of translational and packing entropy terms of Eq. (8), layered structures emerge at high densities where the ideal free energy term is lower for *n* − 1 layers than for *n* ones, while the opposite is true for the excluded distance term. The results of the Onsager theory are shown in Fig. 7. It can be seen that, at the level of the Onsager theory, a first order transition takes place between fluids with 2 and 3 layers for 3 < *H* < 3.5, while only a smooth structural change occurs for 3.5 < *H* < 4. The region corresponding to a first order transition between fluids with 3 and 4 layers extends up to *H* = 4.9, while the structural change shrinks to 4.9 < *H* < 5. In other words, the layering transition becomes stronger with increasing *H*. Regarding the case 2 < *H* < 3, the theory results in a smoothly developing two-layer structure with increasing *η*, without showing any sign of a phase transition. The theory clearly fails in the sense that the packing fraction is not constrained within the close packing limit, i.e. *η* can be higher than *η*_cp_ (the packing fraction at close packing). Therefore it gives unphysical packing fractions which cannot be compared directly with simulation results. However, the trends of the layering transitions coming from the theory and simulations are qualitatively the same.

Returning the attention to the simulation results of the previously studied cases, a couple of relatively minor differences between them are the following. We found that the overall strength of the 3-$\square$ 4-${\diamond }$ transition is larger than the one found for the 1-$\square$ 2-$\hat{\diamond }$ structures. This is observed far from *H*_*c*_, where the transition vanishes for 1-$\square$ 2-$\hat{\diamond }$ but persists for 3-$\square$ 4-${\diamond }$, even for *H* = 4. We may attribute this behaviour to the gain of degrees of freedom on the *y*-axis direction, turning the transition with a more 2d character. As discussed in the previous paragraph, the same behaviour appears for the (*n* − 1)-$\square$ to *n*-$\square$ layering transition. The other one lies on the obtained trends for the right hand side of the phase diagrams. We observe that the (*n* − 1)-$\square$ *n*-$\square$ transition decays faster for the n = 2 case. Note that the trend for *n* = 4 gets nearly parallel to its corresponding maximal packing line after a fast decay, whereas the *n* = 2 transition curve always decays faster than its corresponding maximal packing line. Overall, we can safely state that similarities far exceed differences.

**Figure 8:**
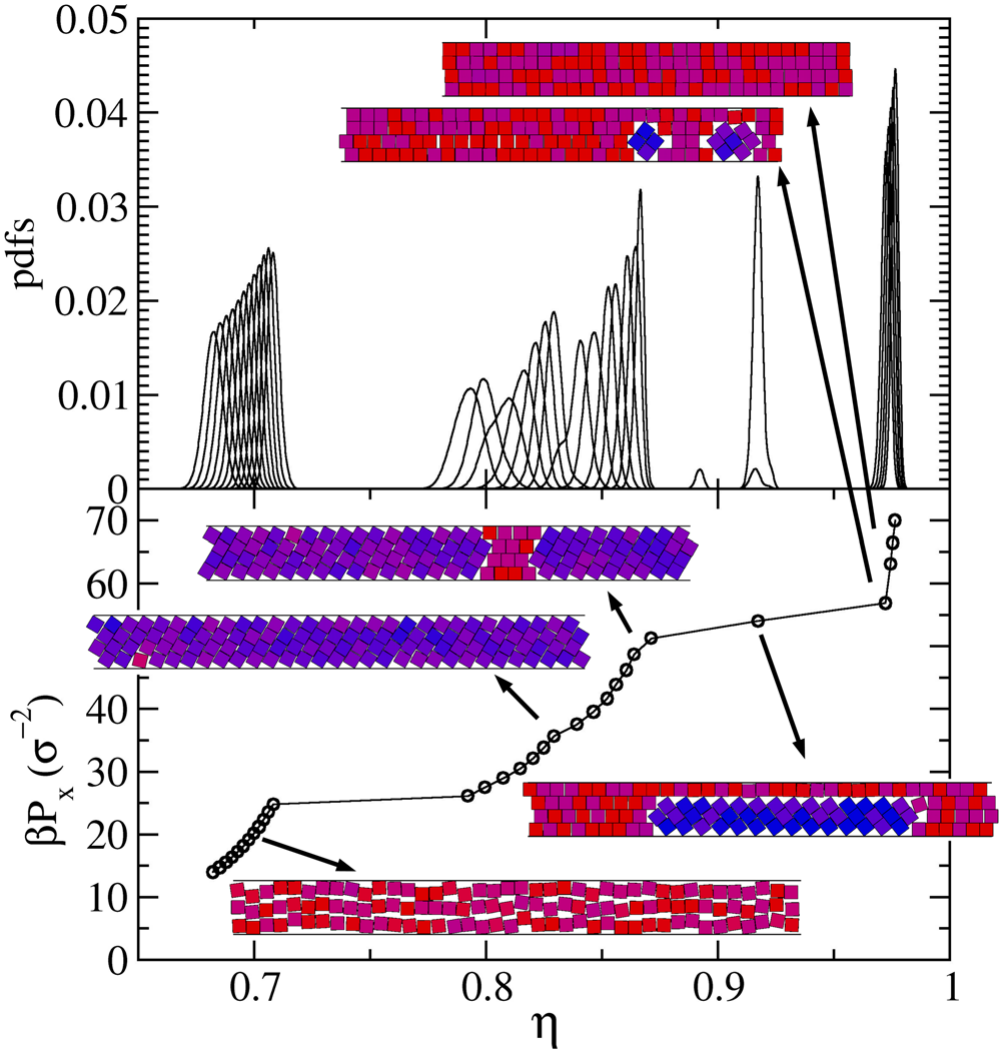
Probability density functions (top) and pressure along the channel (bottom) for *H* = 4.02 and *N* = 120, as obtained by starting from loose and random initial conditions. The approximate locations (replicas continuously swap locations) of the several inserted snapshots are pointed out by the arrows.

To solve the puzzling behaviour for *H* values close to but larger than integer numbers, and in particular around *H* = 4, we performed simulations for *H* = 4.01, 4.02, 4.03 and 4.04. For *H* = 4.04 we simply obtain the 3-$\square$ to 4-$\square$ transition, with larger *η* and *βP*_*x*_ values, following the previously found trend. However, things change for lower *H* values. For *H* = 4.02, and by starting simulations from loose random cells of small systems, we obtain the results given in Fig. 8. There we show the probability density functions (PDFs) and the pressure, *βP*_*x*_, as a function of the packing fraction, *η*. It is observed how the previously found smooth transition, being all PDFs bell shaped and continuously overlapped like the ones given in Fig. 3, splits into three regions, being them clearly separated the one from the other by large gaps. These sets of PDFs lead to the *βP*_*x*_(*η*) function given at the bottom panel of the same figure, which exhibits the three corresponding branches. The left and right ones correspond to the 3-$\square$ and 4-$\square$ phases, respectively. This is confirmed by inspecting several of the replicas appearing at low and high pressure. An inspection of the cells appearing at the middle region yields the elusive (for *H* > 4) 4-${\diamond }$ structure. We also observed several cells having a mixture of structures, suggesting that equilibrium is not reached. This goes in line with the distorted PDFs obtained mostly for the 4-${\diamond }$ region, and with the fact that we have, indeed, never observed a true steady state for the whole set of replicas. The changes are produced very slowly in real time, though, and this is why we, at some point, stopped the simulation. Despite this fact, we are confident the three structures appear, and that the 4-${\diamond }$ competes with the 3-$\square$ and 4-$\square$ structures at low and high pressure, respectively. Hence, we designed two independent runs, one at high pressures with cells filled with 4-${\diamond }$ and 4-$\square$ structures with equal number of particles, and other, at low pressures, with cells having the 4-${\diamond }$ and 3-$\square$ structures. This was done with the aim of determining the transition pressure (as explained in the previous section). Once this is done, we run a short simulation by imposing the expected phases at each pressure range to obtain the transition densities. From them we locate the red bullets of Fig. 6. Nonetheless, much more calculations would be required to do this properly. In particular, the 4-${\diamond }$ to 4-$\square$ transition occurs at high pressures making simulations difficult to carry out.

We would also like to point out here that the mixed structure mentioned in Sec. does frequently appear during the equilibration of the system of replicas for 4 < *H* < 4.03. A snapshot example is given as an input in Fig. 8, which corresponds to the location of the point laying in-between the 4-$${\diamond }$$ and 4-$$\square $$ branches, persisting during the equilibration processes until we stop it. This suggests the mixed structure exhibits a strong metastable behaviour, at least for 4 < *H* < 4.03. Taking into account that $${\eta }_{cp}^{\diamond }$$$$(n)-{\eta }_{cp}^{mix}(n,m)$$ tends to zero for a fixed *m* and *n* → ∞, one can expect it to frequently appear for large *n* values. Indeed, for *n* = 100, H = *n*, and *m* = 1 and 2, $${\eta }_{cp}^{\diamond }$$$$(n)-{\eta }_{cp}^{mix}(n,m)$$ is 2 × 10^−6^ and 4 × 10^−6^, respectively. Besides, one should note that there is a natural tendency for the closest to the wall layer to become parallel, freeing space and letting the rest of the system gain accessible volume and entropy. Hence, although we expect the large *n* phase diagram to behave similarly to those given in Figs 4 and 6, we also expect the appearance of several competing structures showing very strong metastabilities.

**Figure 9:**
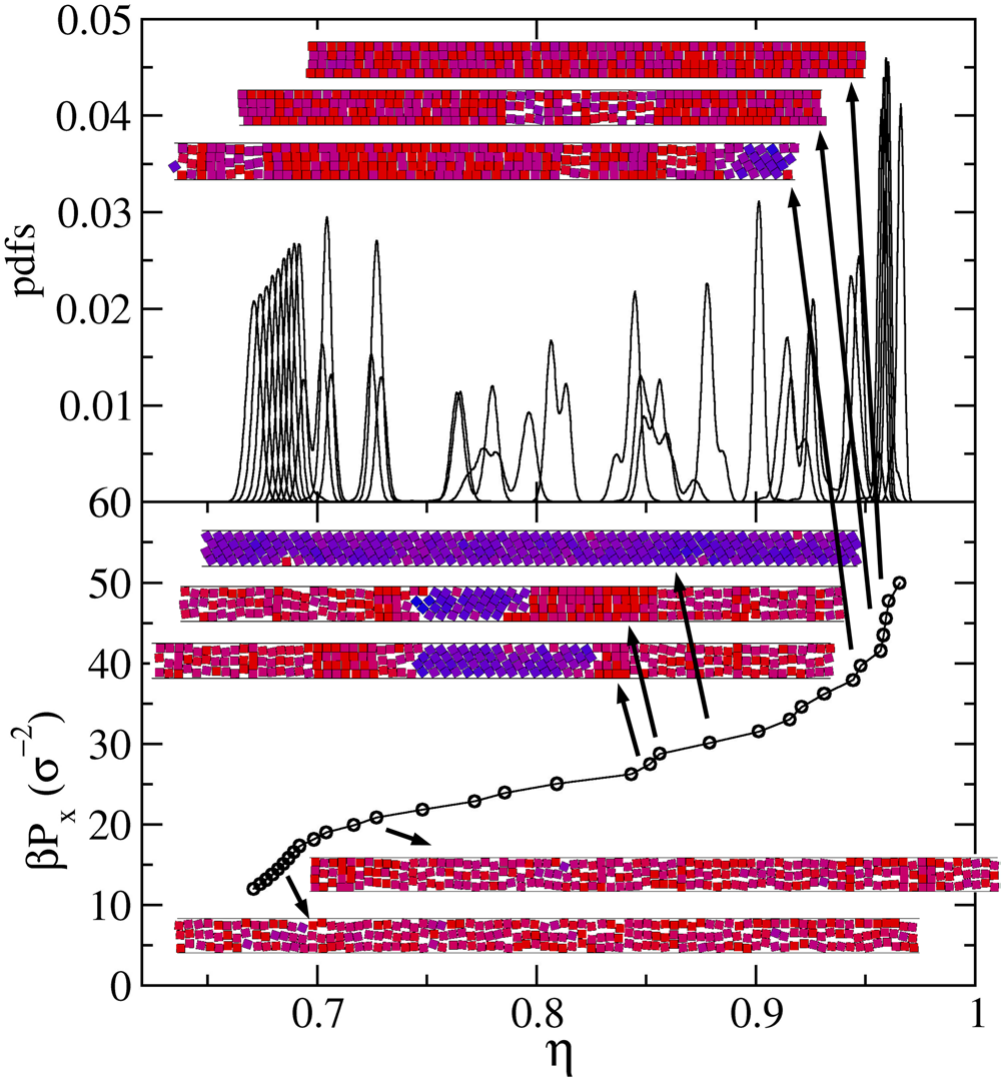
Probability density functions (top) and pressure along the channel (bottom) for *H* = 4.03 and *N* = 120, as obtained by starting from loose and random initial conditions. The approximate location (replicas continuously swap locations) of the several inserted snapshots are pointed out by the arrows.

We should now move to a point in-between *H* = 4.02 and *H* = 4.04, namely *H* = 4.03, to see what happens. It is quite remarkable the sharp change of behaviour occurring in this small *H* range. Figure 9 is built for this intermediate *H* value. At this point, and as it is shown by the snapshots inserted in the figure, we found several microphases. Again, equilibrium, or at least a good equilibrium sampling, is elusive at this point, as can be guessed from the shape of the quite irregular PDFs appearing in between the 3-$\square$ and 4-$\square$ phases at each extreme of the chart. But more importantly, it seems to be a gradual transition from 3-$\square$ to 4-$\square$, passing through several cells containing not only these phases but also the 4-${\diamond }$ one. There is a triple coexistence. Indeed, some single cells have the three coexisting phases. There are, of course, others showing just two phases and some few showing a complete 4-${\diamond }$ structure. There appear several interphases of all possible kinds. That is, there are 3-$\square$ 4-$\square$, 3-$\square$ 4-${\diamond }$, and 4-${\diamond }$ 4-$\square$ interphases. Depending on the *η* location of the replica, it contains more or less of the 3-$\square$ and 4-$\square$ phases, while the 4-${\diamond }$ phase distributes on the entire transition region. We like to conclude this corresponds to a genuine triple point, and place a highlighted red bullet in Fig. 6 at *H* = 4.03.

**Figure 10:**
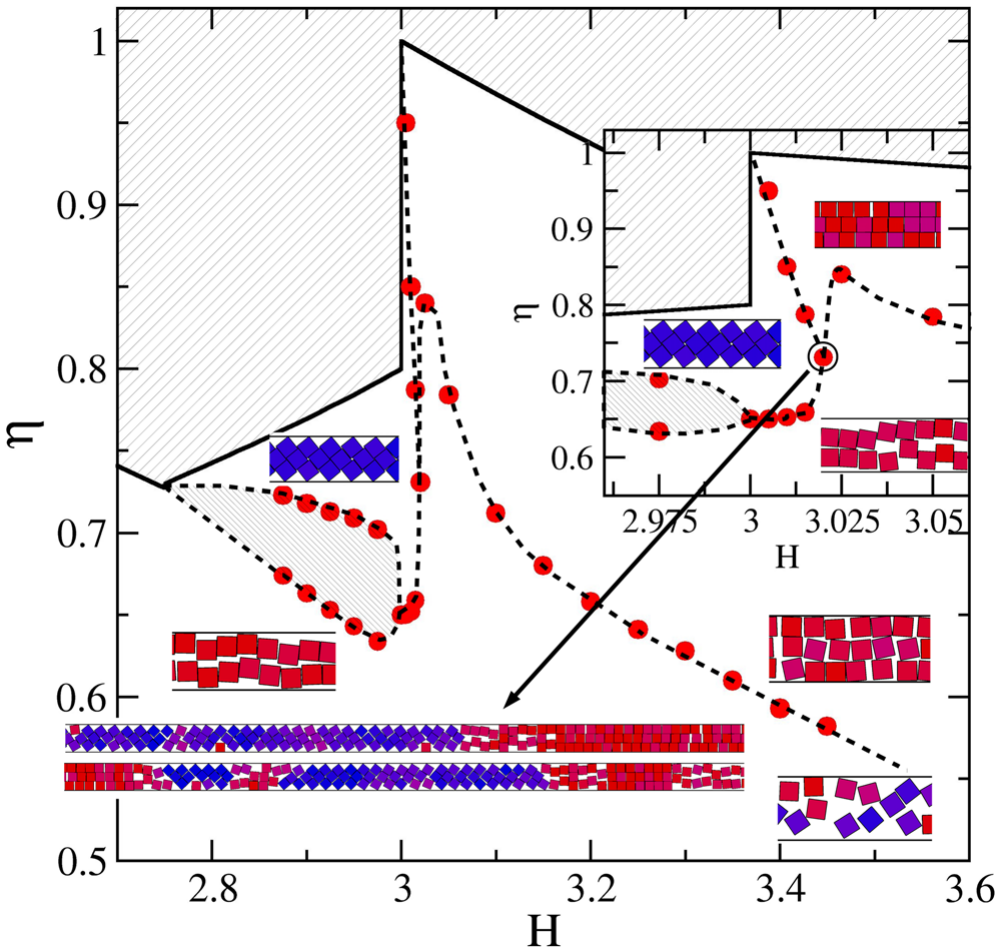
Phase diagram for the *H*_*c*_(3) < *H* < *H*_*c*_(4) region. Red bullets are data from simulations. The inset zooms in the *H* ≈ 3 region. Dashed lines are guides to the eye. Lightly hatched areas are inaccessible. The heavy hatched area points out a transition region in which *P*_*x*_ practically does not depend on *η*. The small snapshots are sections of the simulation cells, which are located according to their *η* and *H* values. The long snapshots pointed by an arrow correspond to cells appearing at the triple point. This point is highlighted by a circle. In the snapshots, parallel and 45-tilted squares are coloured red and blue, respectively. Intermediate angles are painted with a mixture of both colours.

We repeat the whole procedure followed for the *H*_*c*_(4) < *H* < *H*_*c*_(5) range, to obtain the phase diagram corresponding to *H*_*c*_(3) < *H* < *H*_*c*_(4), including a detailed exploration for the pore width of the triple point. The outcome is given in Fig. 10. The resemblance with Fig. 6 is remarkable. We are not listing their similarities; they are too obvious. We should only stress here that we have find a triple point coexistence at *H* = 3.02, i. e. a little bit closer to *H* = 3 than found for *n* = 4. We would also like adding that the strength of the 2-$\square$ 3-${\diamond }$ coexistence lies in-between the *n* = 2 and *n* = 4 cases. In this case the coexistence seems to end at *H* ≈ 2.975.

Finally, we have also performed simulations in the tiny region close to 2, to elucidate the possible existence of a triple point, based on our previous experience with *H* around 3 and 4. We have placed this triple point close to *H* = 2.005.

**Figure 11:**
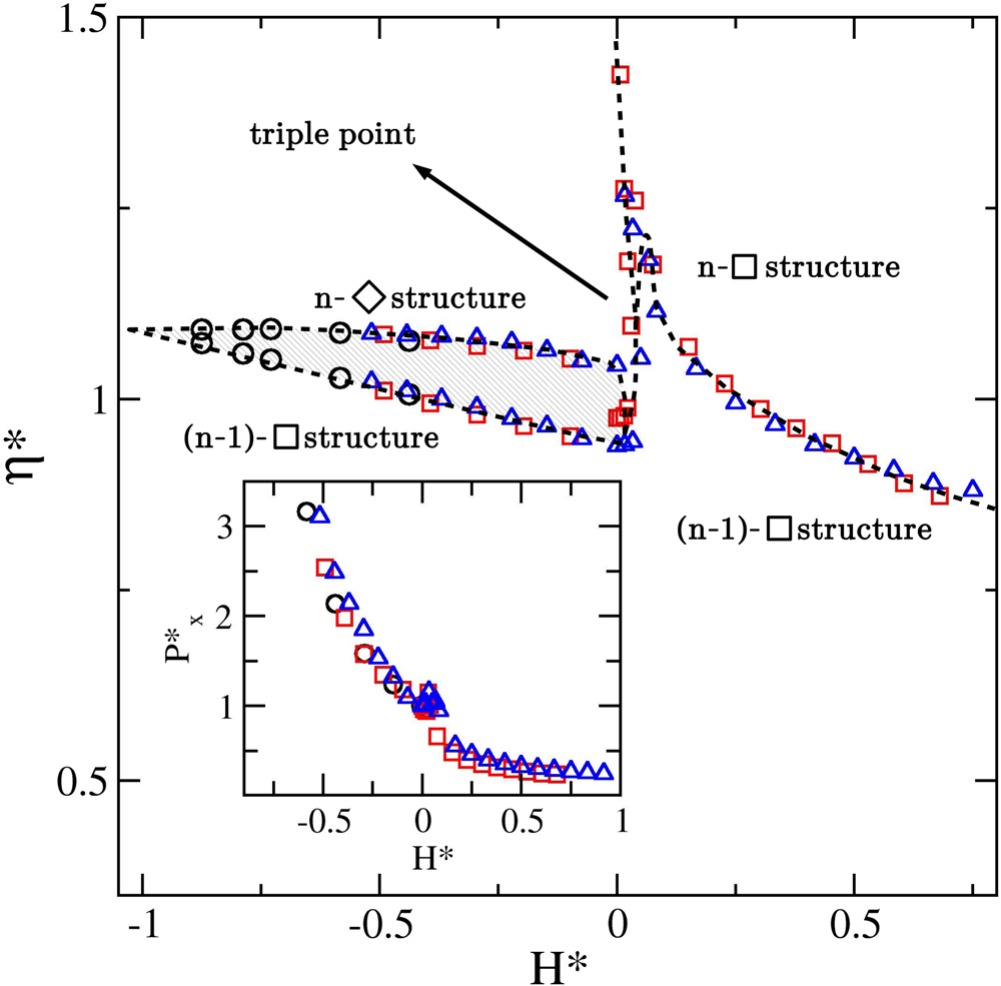
Master phase diagram for a general *H*_*c*_(*n* − 1) < *H* < *H*_*c*_(*n*) range. It is possible to rescale the *η* and *H* axes to yield an approximate collapse of the data for n = 2 (black circles), 3 (red squares) and 4 (blue triangles). See the text for the definitions of η* and H*. The inset shows the approximate collapse of the pressure along the channel by following a similar procedure.

The obtained similarities of Figs 4, 6 and 10, make us wondered if the observed transitions can be unified in a single master phase diagram for all *n* values. By so doing we have found that defining

9 $${H}^{\ast }=\{\begin{array}{ll}(H-n)/(n-{H}_{c}(n)), & {\rm{for}}\,n-1 < H\le n\\ (H-n)/({H}_{c}(n+1)-n) & {\rm{for}}\,n+1\ge H > n\end{array}$$

and ${\eta }^{\ast }=\eta /{\eta }_{cp}^{\square }(n,H=n)$ the desired behaviour approximately arises. The idea of employing the point (${H}_{c}(n),{\eta }_{cp}^{\square }(n,H=n)$) as a reference value comes from the observation that it approximately corresponds to the end of the (*n* − 1)-$\square$ to *n*-${\diamond }$ transition region. The outcome is given in the main panel of Fig. 11, where not all data are shown. On the one hand, we are excluding those with H* < 0 exhibiting no coexistence. On the other hand, we are also excluding those for *n* = 2 and H* > 0, simply because they do not collapse with the others. This is a consequence of the fast drop of *η*(*H*) for 2 < *H* < *H*_*c*_(3), as was already mentioned. We expect data from larger *n* values to behave more likely to the *n* = 3 and *n* = 4 cases. Finally, we are also including, as an inset of Fig. 11, the reduced pressure ${P}_{x}^{\ast }={P}_{x}/{P}_{x}(H=n)$. In this case the data collapse is less striking. It is worth mentioning that this master phase diagram shows clear similarities with the one obtained for slit-like confined 3d-cubes^23^, where our (*n* − 1)-$\square$ and (*n*)-$\square$ structures would correspond to the reported fluid-like and solid-like phases, respectively.

We do not expect Fig. 11 to be precise, and so it should be taken as a guide only. In addition, we may expect differences with the actual phase diagram to enlarge for increasing *n*. We have already mentioned that *H*_*c*_(*n*) → *n* − 1 for *n* → ∞. Hence, at some point, the phase diagram may dramatically change when the *n*-${\diamond }$ structure start competing with the (*n* + 1)-${\diamond }$ structure. We expect this to happen for *n* ≈ 100. Consequently, we speculate that the phase diagram shape given in Fig. 11 could hold for *n* < 100.

## Conclusions

We have built the phase diagram of strongly confined hard squares, for wall-to-wall distances *H* < 4.5, measured in square side length units. The proposed phase diagram includes the maximal packing curve limiting the accessible region for the system. We have observed strong similarities for ranges *H*_*c*_(2) < *H* < *H*_*c*_(3), *H*_*c*_(3) < *H* < *H*_*c*_(4), and *H*_*c*_(4) < *H* < *H*_*c*_(5), where competing structures exhibit clear patterns. This lead us to propose a general phase diagram for *H*_*c*_(*n*) < *H* < *H*_*c*_(*n* + 1), where *H*_*c*_(*n*) refers to a critical point, which coincides with the place at which the maximal packing fraction is achieved by two different structures ((*n* − 1)-$\square$ and *n*-${\diamond }$). Three competing phases fill this master phase diagram, namely (*n* − 1)-$\square$, *n*-$\square$, and *n*-${\diamond }$, which refers to structures formed by *n* − 1 layers of parallel to the walls squares, n layers of parallel squares, and *n* layers of tilted rectangles, each one constituted by n stacked squares. In the case of *n* = 2, the *n*-${\diamond }$ structure is replaced with a zigzag structure, 2-$\hat{\diamond }$. We have also found the presence of triple points, each one corresponding to a different *n*, involving the three mentioned phases. This point is located at $H\gtrsim n$.

One may expect the phase diagram found for slit-like confined 2d-squares to be closely related to that corresponding to slit-like confined cubes. Although work on this direction has been recently carried out^23^, the study fails at high densities as the close packing structures of hard cubes must be similar to that of hard squares. Therefore, there are missing phases in this study.

We can imagine that the close packing structure of our system may be obtained by means of rotational vibration granular experiments, where the production of sharp shapes is much easier than for mesoscale systems. As experimentally found by Zhao *et al*. and corroborated by Avendaño and Escobedo, tiny roundness at square corners can dramatically change the phase diagram of hard squares^6,22^. In our confined system, roundness would work against the parallel structures due to the increasing contribution of the rotational entropy. At certain degree of roundness, we expect the rotor crystalline phases to become more stable than the layered ones. In addition, we also expect the angle of tilted arrangements to increase with increasing roundness. Under this context, our study can be viewed as the limit of small roundness for rounded squares. Finally, certain degree of smoothness of particles and walls can also lead to strong deviations of the hard system behaviour. In the extreme case where only the centres of the squares are confined and there is no orientational restriction, we expect the complete destabilisation of the parallel phases in favour of the tilted structures.
